# TEM in the treatment of recurrent rectal cancer in elderly

**DOI:** 10.1186/1471-2482-13-S2-S56

**Published:** 2013-10-08

**Authors:** Stefano Perrotta, Gennaro Quarto, Vincenzo Desiato, Gianluca Benassai, Bruno Amato, Giacomo Benassai

**Affiliations:** 1 Department of General, Geriatric, Oncologic Surgery and Advanced Technologies, Federico II University, Via S. Pansini 5, 80131, Naples (Italy

**Keywords:** Recurrence of Rectal, Cancer, Mininvasive Surgery, Palliative Surgery

## Abstract

**Introduction:**

Transanal microscopic surgery is an important application of minimally invasive surgery of rectum, allowing realization of complex transanal intervention.

**Patients and Methods:**

During the period between January 2002 and December 2010, seven patients, five men and two women, average age 75 years, with early rectal cancer recurrence were selected for this type of surgical palliative procedure. The selection of the patients is made by: transrectal ultrasonografy, colonoscopy and abdominal ultrasonografy, to rule out liver metastases, CT with and without enema, PET CT. Follow-up is approximately 12-30 months.

**Results:**

The pathologic staging confirms the complete excision of recurrences. Then patients are referred for more complementary therapies.

**Discussion:**

The significance of conservative treatment for local recurrence of rectum adenocarcinoma is still controversial because the recurrence is an expression of tumor spread not controlled by oncological surgical and radio/chemo therapy.

**Conclusion:**

In selected subjects such as the elderly, based on equal oncological treatment, the reduction of surgical trauma, preservation of anatomical integrity and resolution of symptoms are important results.

## Introduction

The microscopic trans-anal surgery, born from an idea by g. Buess, has over 25 years.

Thanks to a continuous improvement of surgical instruments, it's possible realization of complex transanal surgical procedure, like excisions of full thickness resection with anastomotic reconstructions.

Due to the effectiveness achieved, it possible to treat complex cancer cases, in accordance with the improvement of adjuvant and neoadjuvant chemo/radiotherapy. For patients with stage I T1/T2 rectal cancer, local management of rectal cancer has been increasingly considered as an alternative to traditional transabdominal resection. Given the increasing interest in organ and sphincter preservation, LE has rapidly gained appeal. However, its oncologic adequacy remains controversial [1-5].

In our experience, we have extended the use of TEM in palliative treatment of adenocarcinoma recurrence after surgery in the elderly, where the resolution of the symptoms, like bleeding and/or sub-occlusion, the maximization of survival benefit, minimization of disease recurrence, and preservation of preoperative bowel function are acceptable results in select cases, allowing you to avoid overly demolition surgery, especially in relation to age-related comorbidities.

## Patients and Methods

the instruments used is composed of a operator rectoscope of 4 cm diameter with variable length from 12 to 20 cm, and operating 4 channels.

The sigmoidoscope is fixed to operating table with mechanical arm while a combined endosurgery unit delivers CO2 in rectum to obtain a dilation at a constant pressure of 12-14 mmHg. The HD optic provides a three-dimensional view and play back images on the monitor.

In the period between January 2010 and December 2012, 7 patients, 5 males and 2 females, average age 75 years, have undergone minimally invasive surgical treatment for recurrent adenocarcinoma of rectum.

4 males and 1 female were subjected to ultra-low anterior resection of rectum adenocarcinoma followed by cycles of adjuvant chemo/radio therapy.

The lesions were assessed as T3N1M0 at histological examination.

1 woman had been subjected to TEM as first procedure for a lesion evaluated as T1NxM0 and in 1 male patient, 78 years old, local excision was performed after cycles of neoadjuvant chemo/radio terapy for a T2NxM0 lesion. During the follow-up, approximately 22-28 months after first surgery, cancer recurrence is presented.

In this period patients underwent blood tests to check in particular cancer markers, half-yearly CT Total Body to assess lymph node and metastases and annual colonoscopy to evaluate possible recurrence and/or new colonic lesions. In all patients recurrence is contained in rectum at level of the rear wall and less than 2 cm in diameter (evaluation obtained by execution of trans rectal ultrasonografy at identification of new lesions).

In all cases the subjects have refused major surgery in relation to 'high risk intra-, peri-and postoperative mortality related to age-related comorbilities present.

## Results

the mean operating time is 50 min (range 10-110) and blood loss is < 50 ml in all patients. Histological results confirms the complete excision of recurrence. Two patients experience postoperative complications. One develops acute urinary retention. One patients develops pelvic sepsis, for which a defunctioning ileostomy is formed and subsequently reversed. There are no procedure-related deaths. The median hospital stay is 4 days (range 2-14). Follow up is approximately 12-30 months. Patients are referred then for more complementary therapies. Two patients have developed distant metastases. One patients with T3 carcinoma develops brain metastases at 30 months post TEM and died. The other patient develops liver metastases 25 months after TEM for a T2 carcinoma.

## Discussion

Radical surgery with mesorectal excision is considered the oncological standard in the curative treatment of rectal cancer. Independent of the cancer stage, the complication rates of elective RS are reported to be as high as 30-40% (including impotence and urological dysfunction) with a mortality rate of 2-5% and a definitive colostomy is required in approximately 30% of these patients [6-9]. Compared with RS, the main advantage of limited surgical intervention is the appreciably lower morbidity and mortality rates and the better functional results. As previously pointed out, preoperative staging is a challenge, even using the best available techniques, but due to the risk of lymph node metastases, preoperative correct definition of the lesion's extension is mandatory to avoid unnecessary major surgery and complications.

Although some authors included tumor size in the preoperative selection criteria, we understood there were no conclusive data to support this point and, instead of the size by itself, the inclusion criteria was the tumor suitability for getting adequate free margins. More recently neoadjuvant or adjuvant radiotherapy with or without chemotherapy has been used to improve local recurrence rates following local excision of rectal cancers. Local recurrence for T2-3, N0 rectal tumours after TEM with neoadjuvant chemoradiotherapy is reported to be comparable to that following laparoscopic resection. Reduction in tumour size after neoadjuvant chemoradiotherapy was found to be the most reliable prognostic factor of success of local excision [10,11].

The significant differences between LE and SR as described earlier constitute the fundamental consideration when assessing the oncologic adequacy of LE versus SR in T1/T2 tumors.

First there is evidence that tumor extension in the bowel wall distal to the palpable tumor edge is relatively uncommon. Second LE does not aim to remove the mesorectum, whereas SR aims to remove the entire mesorectal package. The general incidence of occult nodal involvement in T1 tumors ranges from 10% to 13%. The rate increases to at least17%to22%for T2 tumors. Same predictive models were developed to predict nodal involvement based on T stage and other factors including patient age, tumor histology, degree of differentiation, lymphocytic infiltration, and evidence of vascular or perineural invasion. Third, preoperative selection of patients for TEM or SR depends on the accuracy of clinical staging by preoperative imaging [12-16]. Reviewed in detail elsewhere, the preoperative staging of rectal adenocarcinoma has significantly improved but remains imperfect. The most commonly used imaging modalities include endoscopic ultrasound (EUS) and magnetic resonance imaging (MRI). The reported stage-specific sensitivities and specificities of EUS are T1 (88% and 98%), T2 (81% and 96%), T3 (96% and 91%), and T4 (95% and 98%); corresponding sensitivity and specificity for nodal staging are 73% (95% confidence interval, 71%-76%) and 76% (95% confidence interval, 74%-78%), respectively. Finally, for patients who undergo LE but later develop a recurrence in the pelvis, salvage operations typically involve multivisceral pelvic resections, with morbidity rates of 34% and R0 resection rates between 79% and 94%. The 5-year disease-free survival after salvage surgery ranges between 53% and 59% at best. By contrast, for patients who undergo LE but were found to have either a T3 lesion, evidence of lymphovascular invasion, or gross residual disease on pathology, proceeding to immediate salvage resection does not appear to compromise long-term outcomes, with reported 5-year overall survival rates of 79% [17-21]. Thus, the risk of delayed failure after LE may be costly and the importance of vigilant tumor surveillance after resection underscored. Current evidence suggest that local surgical excision may be considered as an alternative to SR only in very select few patients whose disease is confined and tumor biology is highly favourable [22-25]. In the current era of personalized medicine, the optimal treatment plan for an individual patient requires a well-informed discussion. A careful consideration of multiple key factors would inform an individualized analysis of benefits and risks of LE versus SR [17-20]. New approaches in multimodality therapy aimed at improving oncologic outcome after LE alone have emerged but remain in the setting of clinical trials [26-28].

## Conclusions

Recurrence of rectal cancer constitute a failure to control by the previous surgical procedure. The subsequent surgical approach after ultra low resection is abdominal perineal resection sec Milles. This complex procedure is to be burdened not only by complicaze like bleeding, urinary disorders, abscesses, etc., but it is also a procedure extremely disability due to permanent loss of sphincter function. In addition, this procedure may not be oncologically safe for possibility of lymph node micrometastases not properly evaluated and controlled. For this reason, in selected subjects such as the elderly, TEM may be a possible alternative to radical surgery where the resolution of symptoms such as bleeding, sub-occlusion can be a successful in relation to high-risk surgical and anesthetic related to co-morbidity age related.

## Authors' contributions

S.P.: conception and design, interpetration of data, given final approval of the version to be published.

G.Q: critical revision, interpretation of data, given final approval of the version to be published

V.D.: acquisition of data, drafting the manuscript, given final approval of the version to be published

G.L.B.: acquisition of data, drafting the manuscript, given final approval of the version to be published

B.A.: critical revision, interpretation of data, given final approval of the version to be published

G.Q: critical revision, interpretation of data, given final approval of the version to be published

G.B.: conception and design, critical revision, given final approval of the version to be published

## Authors' information

SP: Resident in Surgery at University Federico II of Naples.

GQ: Assistant Professor in Surgery at University Federico II of Naples.

VD: Resident in Surgery at University Federico II of Naples.

GLB: Resident in Cardiology at University Federico II of Naples.

BA: Associate Professor of Surgery at University Federico II of Naples.

GB: Assistant Professor in Surgery at University Federico II of Naples.
